# Mechanical transmission of SARS-CoV-2 by house flies

**DOI:** 10.1186/s13071-021-04703-8

**Published:** 2021-04-20

**Authors:** Velmurugan Balaraman, Barbara S. Drolet, Dana N. Mitzel, William C. Wilson, Jeana Owens, Natasha N. Gaudreault, David A. Meekins, Dashzeveg Bold, Jessie D. Trujillo, Leela E. Noronha, Juergen A. Richt, Dana Nayduch

**Affiliations:** 1 Department of Diagnostic Medicine/Pathobiology and Center of Excellence for Emerging and Zoonotic Animal Diseases, College of Veterinary Medicine, Kansas State University, 1800 Denison Ave, Manhattan, KS 66506 USA; 2 Arthropod-Borne Animal Diseases Research Unit, Department of Agriculture, Agricultural Research Service, 1515 College Ave, Manhattan, KS 66502 USA

**Keywords:** SARS-CoV-2, Coronavirus, House flies, Transmission, Mechanical vector

## Abstract

**Background:**

Severe acute respiratory syndrome coronavirus 2 (SARS-CoV-2) is a recently emerged coronavirus that is the causative agent of the coronavirus disease 2019 (COVID-19) pandemic. COVID-19 in humans is characterized by a wide range of symptoms that range from asymptomatic to mild or severe illness including death. SARS-CoV-2 is highly contagious and is transmitted via the oral–nasal route through droplets and aerosols, or through contact with contaminated fomites. House flies are known to transmit bacterial, parasitic and viral diseases to humans and animals as mechanical vectors. Previous studies have shown that house flies can mechanically transmit coronaviruses, such as turkey coronavirus; however, the house fly’s role in SARS-CoV-2 transmission has not yet been explored. The goal of this work was to investigate the potential of house flies to mechanically transmit SARS-CoV-2. For this purpose, it was determined whether house flies can acquire SARS-CoV-2, harbor live virus and mechanically transmit the virus to naive substrates and surfaces.

**Methods:**

Two independent studies were performed to address the study objectives. In the first study, house flies were tested for infectivity after exposure to SARS-CoV-2-spiked medium or milk. In the second study, environmental samples were tested for infectivity after contact with SARS-CoV-2-exposed flies. During both studies, samples were collected at various time points post-exposure and evaluated by SARS-CoV-2-specific RT-qPCR and virus isolation.

**Results:**

All flies exposed to SARS-CoV-2-spiked media or milk substrates were positive for viral RNA at 4 h and 24 h post-exposure. Infectious virus was isolated only from the flies exposed to virus-spiked milk but not from those exposed to virus-spiked medium. Moreover, viral RNA was detected in environmental samples after contact with SARS-CoV-2 exposed flies, although no infectious virus was recovered from these samples.

**Conclusions:**

Under laboratory conditions, house flies acquired and harbored infectious SARS-CoV-2 for up to 24 h post-exposure. In addition, house flies were able to mechanically transmit SARS-CoV-2 genomic RNA to the surrounding environment up to 24 h post-exposure. Further studies are warranted to determine if house fly transmission occurs naturally and the potential public health implications of such events.

**Graphical abstract:**

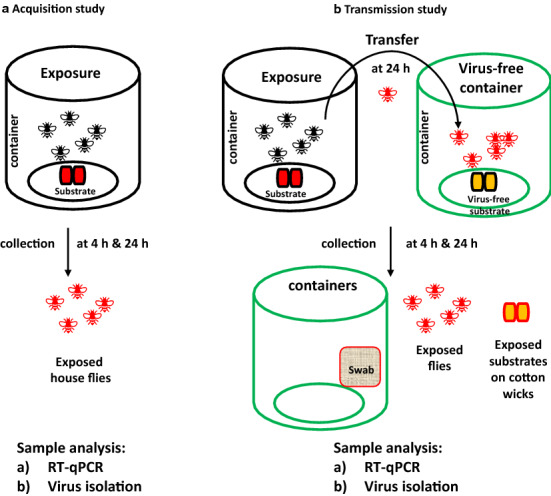

**Supplementary Information:**

The online version contains supplementary material available at 10.1186/s13071-021-04703-8.

## Background

Severe acute respiratory syndrome coronavirus 2 (SARS-CoV-2) is the causative agent of the ongoing coronavirus disease 2019 (COVID-19) pandemic. This virus was first reported in Wuhan, China, in 2019 [1] and has since spread worldwide. To date, the virus has spread to over 219 countries and territories, infected more than 133 million people and caused approximately 2.9 million deaths globally (Coronavirus Resource Center, John Hopkins: https://coronavirus.jhu.edu/map.html, accessed 8th April 2021). SARS-CoV-2 is an enveloped, positive-sense, single-stranded RNA virus that belongs to the betacoronavirus genus. It infects humans and a number of domestic and wild animals [2–4]. Previous studies have shown that the virus is highly contagious and stable in the environment for extended periods [5, 6]. Human-to-human transmission occurs* via* the oral-nasal route by inhalation of infectious droplets or aerosols or contact with contaminated surfaces [5].

Arthropods play a significant role in both the mechanical and biological transmission of a number of viral pathogens [7]. In biological transmission, the virus infects and replicates in the arthropod vector and is subsequently transmitted to susceptible hosts during feeding (e.g. arbovirus transmission by mosquitoes). In the case of mechanical transmission, the virus is passively acquired and transiently harbored internally or on the external surfaces of the vector and then transported from source to source (e.g. pathogen transmission by filth flies). Several recent studies have explored the potential for hematophagous arthropods to be a vector for SARS-CoV-2 [8–12]. House flies can harbor over 250 different pathogens, such as bacteria, viruses, protozoa, helminths and fungi [13]. They are attracted to humans and animal exudates and excreta, as well as human and animal food items, which provides potential pathogen acquisition opportunities and transmission routes [13]. House flies easily move between contaminated and clean environments and disseminate microbial agents in the process. Numerous studies have demonstrated that house flies can acquire and harbor pathogens both on their exterior surfaces (e.g. wings, legs, mouthparts) and internally in their alimentary canal [13, 14]. Moreover, house flies have been demonstrated to transmit coronaviruses, namely the turkey coronavirus (TCV) [15], and other important viral agents, including Newcastle disease virus [16], porcine respiratory syndrome virus [17] and porcine transmissible gastroenteritis [18]. However, the potential for house flies to acquire, harbor and transmit SARS-CoV-2 has not been investigated yet.

In this study, we examined the potential for house flies to mechanically aquire, harbor and transmit SARS-CoV-2. Understanding this potential role of house flies in the ecology of SARS-CoV-2 transmission is important for assessing the risk to public health and implementing the respective mitigation strategies.

## Methods

### Virus and cells

Vero E6 cells (ATCC® CRL-1586™; American Type Culture Collection, Manassas, VA, USA) were used for the cultivation and titration of SARS-CoV-2. Cells were maintained in Dulbecco’s Modified Eagle’s Medium (DMEM; Corning, New York, NY USA), supplemented with 5% fetal bovine serum (R&D Systems, Minneapolis, MN, USA) and antibiotics/antimycotics (Thermo Fisher Scientific, Waltham, MA, USA), called DMEM growth medium, at 37 °C under a 5% CO_2_ atmosphere in a cell culture incubator. The SARS-CoV-2 USA-WA1/2020 strain was acquired from the Biodefense and Emerging Infection Research Resources Repository (BEI Resources, Manassas, VA, USA) and was passaged three times on Vero E6 cells with a final titer of 7 × 10^5^ 50% tissue culture infective dose (TCID50)/ml.

### Preparation of test substrates

The stability of SARS-CoV-2 was tested in the following house fly feeding substrates: egg yolk powder (Bulkfoods.com, Toledo, OH, USA), non-fat milk powder (GreatValue; Walmart Inc., Bentonville, AR, USA) and powdered sugar (GreatValue, Walmart). A 10% (w/v) virus-spiked substrate was prepared by mixing each substrate with virus in DMEM growth medium. Additionally, a virus-spiked medium (positive control) was prepared by adding virus to DMEM growth medium. All virus-spiked substrates and the positive medium control contained a final SARS-CoV-2 concentration of 5 × 10^4^ TCID_50_/ml. DMEM growth medium with no virus served as the negative control.

### Stability of SARS-CoV-2 in various substrates

Ten individual cotton wicks (approx. 0.25 cm^3^ in size) were placed into separate wells of four different 12-well tissue culture plates (Corning). Two ml of three different substrates were added to six wicks and 2 ml of control substrate was added to four wicks. The final concentration of virus was 10^5^ TCID_50_ per well. The plates were incubated in an environmental chamber at 22 °C and 60% relative humidity. The substrates were collected by adding 1 ml of DMEM growth medium onto the wicks and then, using a micropipettor, the medium was pipetted up and down 5 times before collection. The samples were collected at 0, 1, 4 and 24 h post-inoculation from the plates and stored at − 80 °C until analyzed. The virus titer of each sample was determined by performing the TCID_50_-cytopathic effect (CPE) assay (see below).

### House fly maintenance

House flies (*Musca domestica*) were maintained at the U.S. Department of Agriculture, Center for Grain and Animal Health Research, Arthropod-Borne Animal Diseases Research Unit (ABADRU-USDA). Female house flies (1–2 days old, unmated) were fasted and water was removed for 12–16 h prior to exposure to the feeding substrate. On the day of the study, flies (*n* = 60) were anesthetized with CO_2_ for 1–2 s and placed into 12 individual polypropylene containers (*n* = 5/container), approximately 250 ml in size. Each container contained two cotton wicks placed in a small weigh boat and the container was sealed with a mesh lid to prevent fly escape while allowing air circulation. Primary containers were placed in a secondary container and were transported to the Biosecurity Research Institute (BRI) at Kansas State University for studies under Arthropod Containment Level-3 (ACL-3) conditions.

### Virus acquisition by house flies

**Fig. 1:**
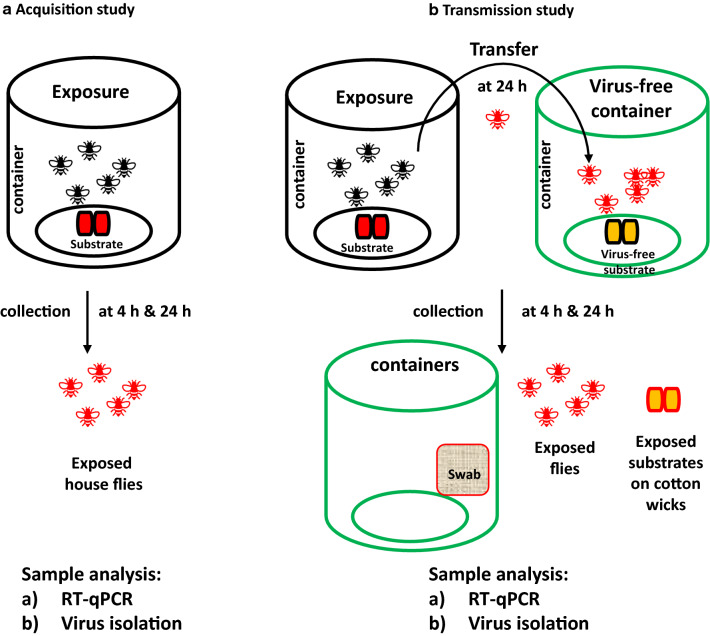
House fly mechanical transmission of SARS-CoV-2 study design. **a** Acquisition study: house flies were exposed to virus-spiked substrate or positive and negative controls on wicks for 4 and 24 h, following which the flies were collected. **b **Transmission study: house flies were exposed to the same set of substrates on wicks for 24 h and transferred to a second container containing virus-free substrate on wicks. At 4 and 24 h post-transfer, the flies, swabs and exposed substrate on wicks were collected. Sample analysis: the  flies, container inner surface swabs and substrate samples collected from the studies were processed and tested for SARS-CoV-2 nucleocapsid gene by quantitative reverse transcription-PCR (*RT-qPCR*) and for infectious virus by virus isolation

House flies were anesthetized by exposure to cold (− 20 °C for 5 min), then one of the three feeding substrates was introduced to four fly containers each by pipetting 2 ml onto the cotton wicks. The feeding substrates were: (i) SARS-CoV-2 in 10% non-fat milk feeding substrate; (ii) SARS-CoV-2 in DMEM growth medium (positive control); or (iii) DMEM growth medium without virus (negative control). The final concentration of SARS-CoV-2 in the spiked feeding substrate and in the positive control was 1 × 10^5^ TCID_50_. At 4 h post-exposure, two containers from each of the three groups were placed at − 20 °C for 30 min to sacrifice the flies. Then, individual flies were collected and placed in tubes with 1 ml transport medium (199E medium, 200U/ml penicillin, 200 µg/ml streptomycin, 100 µg/ml gentamycin and neomycin, 5 µg/ml amphotericin B; Sigma-Aldrich, St. Louis, MO, USA). Transport medium was used for storage and as a processing medium for the collected samples. Container swabs were collected by wiping the interior surface of the containers with a sterile cotton swab soaked in 1 ml of transport medium; they were re-immersed into the same transport medium for further evaluation. This procedure was repeated on the remaining two containers from each group after 24 h exposure (Fig. 1). The collected flies and container swabs were stored at − 80 °C until further processing for infectious virus and viral RNA.

### Virus transmission by house flies

A second set of house flies (*n* = 60) were fed with the three feeding substrates as described above for a 24 - time period. Exposed flies were then transferred to a new set of 12 containers containing 2 ml of virus-negative test substrate, i.e. 10% non-fat milk powder in growth medium on cotton wicks. The flies were kept for 4 or 24 h post-transfer. At each time point, the flies, the substrate and the inner surface of the containers were swabbed and collected as described above and the swabs and flies stored at − 80 °C until further processing.

### Sample processing

Each fly collected during the experiments described above was homogenized using a Tissuelyser II (Qiagen, Germantown, MD, USA). Briefly, individual flies were placed in a 1.5-ml microcentrifuge tube containing one sterile 3-mm tungsten carbide bead (Qiagen) and 1 ml of virus transport medium. The flies were homogenized for 30 cycles for 3 min. Homogenates were filtered through a 0.22-µm polyethersulfone (PES) membrane filter (MIDSCI, St. Louis, MO, USA). The collected swabs were removed from the tubes with the transport medium and then filtered as descibed above.

### RNA extraction and quantitative reverse transcription-PCR

RNA was extracted from 50 µl of filtered house fly homogenate, 140 µl of filtered swab medium and 140 µl of the respective feeding substrate solution using the QIAamp Viral RNA Mini Kit (Qiagen) according to the manufacturer’s instructions. The quantitative reverse transcription-PCR (RT-qPCR) assay was performed according to the RT-qPCR protocol established by the US Centers for Disease Control and Prevention (CDC) for the detection of the SARS-CoV-2 nucleocapsid (N)-specific RNA (https://www.fda.gov/media/134922/download) using the N2 primer and probe set and the qScript XLT One-Step RT-qPCR Tough Mix (Quanta Biosciences, Beverly, MA, USA) on a CFX96 real-time thermocycler (Bio-Rad, Hercules, CA, USA). The PCR plate controls included a quantitated SARS-CoV-2 N-specific qPCR positive control, diluted 1:10 (Integrated DNA Technologies, Coralville, IA, USA), and a non-template control (NTC). The results were analyzed using Bio-Rad CFX Manager 3.1 software. Samples with Cq values of < 38 were considered positive for SARS-CoV-2 RNA. A reference standard curve method using  SARS-CoV-2-specific RNA as a standard was used for calculating the SARS-CoV-2 copy number of each sample, as previously described [3, 19].

### Virus isolation

Vero E6 cells were plated at a density of 1 × 10^4^ cells in 100 µl medium per well in 96-well plates. After overnight incubation, 50 µl of filtered fly homogenates or 50 µl of filtered swab solution was pipetted into each well in duplicate. Feeding substrates were diluted 1:10 in transport medium and filtered as described above,  then 50 µl was pipetted into duplicate wells. Every sample was blind passaged three times at 3 days post-infection (dpi) by transferring approximately 150 µl of the culture medium from each well onto a new Vero E6 monolayer in 96-well plates.

### Immunofluorescent assay 

After the first and third passage, Vero E6 monolayers were fixed and stained with mouse monoclonal antibodies specific for the SARS-CoV-2 RBD region and a fluorescein-labeled goat anti-mouse antibody as previously described [11]. Monolayers were examined for fluorescein isothiocyanate-positive cells with an EVOS fluorescent microscope (Thermo Fischer Scientific, Waltham, MA, USA). Mock-infected and SARS-CoV-2 infected Vero E6 cells were used as negative and positive controls, respectively, as shown in Additional file 1: Figure S1.

### TCID_50_-CPE assay

To determine virus titer, each sample was tenfold serially diluted in DMEM growth medium and added onto Vero E6 cell monolayers in 96-well plates. The  inoculated wells were observed for the presence of CPE after 3–5 dpi. The virus titer of each sample was calculated using the Spearman–Karber method [21].

## Results

### Virus stability in feeding substrate

To determine a suitable substrate for virus acquisition in house flies, virus stability in different fly feeding substrates was tested. A dose of 10^5^ TCID_50_ SARS-CoV-2 was added to 10% solutions of egg yolk powder, non-fat milk powder and sugar powder, respectively, and the titer of infectious virus was tested over a period of 24 h. The SARS-CoV-2 titer was determined to be most stable in the 10% milk solution, with only a 0.52 log_10_ decrease in virus titer over 24 h, followed by the sugar and the egg yolk solution (Additional file 2: Table S1). Therefore, the 10% non-fat milk powder was chosen as a substrate for both the acquisition and transmission studies.

### Acquisition of SARS-CoV-2 by house flies

To determine whether house flies could aquire and harbor SARS-CoV-2, 20 flies each were housed in containers with either medium or milk substrate spiked with 1 × 10^5^ TCID_50_ of SARS-CoV-2, or with virus-free medium (Fig. 1a). After 4 and 24 h of exposure, ten flies were removed from each container and homogenized individually to determine if SARS-CoV-2 RNA could be detected. SARS-CoV-2 viral RNA was detected on all ten flies at both 4 and 24 h post-exposure in the virus-spiked medium and the milk-feeding substrate group. At 4 h post-exposure, both groups had 2.1 × 10^6^ and 3.1 × 10^6^ copy number (CN) of SARS-CoV-2 N-specific RNA/µl of homogenate, respectively. The level of SARS-CoV-2 RNA increased to 3.8 × 10^6^ CN/µl in the milk-feeding substrate group at 24 h post-exposure, but decreased to 4.1 × 10^5^ CN/µl in the virus-spiked medium control group. No viral RNA was detected in any of the flies at 4 or 24 h post exposure in the virus-free negative control group. In addition to RT-qPCR analysis for the presence of viral RNA, the fly homogenate samples were plated onto Vero E6 cells for virus isolation (VI) in order to determine if infectious virus could be detected in the house fly samples. No infectious virus was detected in any of the negative control house flies, or in flies exposed to SARS-CoV-2-spiked medium (Table 1). However, infectious virus was recovered from 20% of the flies exposed to the milk substrate spiked with SARS-CoV-2 at both 4 and 24 h post-exposure. These results indicate that house flies are indeed capable of aquiring SARS-CoV-2 from medium or milk substrates spiked with SARS-CoV-2. In addition, house flies are able to harbor infectious virus when exposed to milk substrate spiked with SARS-CoV-2.

**Table 1:** Acquisition study: Detection of SARS-CoV-2 viral RNA and infectious virus in flies

Substrate group	4 h of exposure		24 h of exposure	
	PCR+, flies/*N* (copy number/µl of sample)	VI /IFA+, flies/*N*	PCR+, flies/*N *(copy number /µL of sample)	VI/ IFA+, flies/*N*
Medium only (negative control)	0/10 (ND)	0/10	0/10 (ND)	0/10
Virus-spiked medium (positive control)	10/10 (2.1 × 10^6^)^a^	0/10	10/10 (4.1 × 10^5^)^a^	0/10
Virus-spiked milk	10/10 (3.1 × 10^6^)^b^	2/10	10/10 (3.8 × 10^6^)^b^	2/10

IFA, Immunofluorescent assay; *N*, total number of flies; ND, not detected; VI, virus isolation

^a^Standard deviation (SD) of virus-spiked medium group flies at 4 and 24 h exposure: 2.8 × 10^6^ and 1.6 × 10^5^, respectively

^b^SD of virus-spiked milk group flies at 4 and 24 h exposure: 1.8 × 10^6^ and 2.2 × 10^6^, respectively

### Mechanical transmission of SARS-CoV-2 by house flies

To gain insights into whether flies are able to transmit SARS-CoV-2 virus to their immediate environment, the inside surface of the containers holding flies fed with different substrates were swabbed and the swabs tested for the presence of viral RNA and/or infectious virus. Viral RNA was detected on the inside container surface from the container with flies fed medium and milk spiked with SARS-CoV-2 at both 4 and 24 h post-exposure (Additional file 3: Table S2). In addition, infectious virus was detected in one of two swabs collected from containers with flies fed the SARS-CoV-2-spiked medium at 4 h post-exposure. No infectious virus was detected in any of the other samples, including the negative control. These results indicate that SARS-CoV-2 exposed flies are indeed capable of spreading infectious virus within their immediate environment.

### Transmission of SARS-CoV-2 by house flies to substrates and surfaces

To gain further insights into SARS-CoV-2 transmission by flies, an additional study was performed to determine whether house flies could transmit acquired virus to a virus-free substrate and container surfaces (Fig. 1b). As in the previous study, all flies were exposed to either SARS-CoV-2-positive or -negative medium or virus-spiked milk for 24 h. Flies were then transferred to new containers and a virus-free substrate was added to wicks (10% milk in DMEM growth medium) for 4 and 24 h. The substrates and the inside of the container were then examined for any contaminating viral RNA or infectious virus. Viral RNA was detected in all virus-exposed flies at both 4 and 24 h post-transfer (Tables 2, 3), and infectious virus was detected in 40% and 10% of flies exposed to the SARS-CoV-2-spiked milk at 4 and 24 h post-transfer, respectively; this result indicates that the SARS-CoV-2-exposed flies did harbor SARS-CoV-2  up to 24 h after transmission to virus-free substrates and surfaces. After 4 and 24 h post-transfer, SARS-CoV-2 RNA was detected on all virus-free transfer containers that held both groups of virus-exposed flies (Tables 2, 3). The positive medium control group transfer container had 7.2 × 10^3^ CN of viral RNA/µl at 4 h post-transfer, which increased to 8.7 × 10^4^ CN/µl at 24 h post-transfer (Tables 2, 3). A similar trend in CN increase over time was observed for transfer containers exposed to flies fed with virus-spiked milk substrate, with 2.6 × 10^4^ CN of viral RNA/µl at 4 h post-transfer and 1.1 × 10^5^ CN/µl at 24 h post-transfer. To further investigate if infectious virus was present in the transfer container swabs, the samples were blind passaged on Vero E6 cells. However, none of the swabs from the transfer containers tested positive for infectious virus.

**Table 2:** Transmission study: Detection of SARS-CoV-2 viral RNA and infectious virus in substrates, transfer containers and flies at 4 h post-transfer

Substrate group	PCR+, substrate/*N* (copy number/µl of sample)	PCR+, container surface/*N* (copy number/µl of sample)	PCR+, flies/*N* (copy number/µl of sample)	VI /IFA+, flies/*N*
Medium only (negative control)	0/2 (ND)	0/2 (ND)	0/10 (ND)	0/10
Virus-spiked medium (positive control)	2/2 (9.3 × 10^1^)^a^	2/2 (7.2 × 10^3^)^a^	10/10 (3.9 × 10^6^)^a^	0/10
Virus-spiked milk	2/2 (1.1 × 10^2^)^b^	2/2 (2.6 × 10^4^)^b^	10/10 (2.3 × 10^7^)^b^	4/10

*N *, Total number of samples (wicks, flies or swabs); ND, not detected

^a^SD of virus-spiked medium group substrate, swabs and flies at 4 h post-transfer: 7.5 × 10^1^, 6.7 × 10^3^ and 6.0 × 10^6^, respectively

^b^SD of virus-spiked milk group substrate, swabs and flies at 4 h post-transfer: 2.0 × 10^1^, 4.7 × 10^3^ and 1.2 × 10^7^, respectively

**Table 3:** Transmission study: Detection of SARS-CoV-2 viral RNA and infectious virus in substrates, transfer containers and flies at 24 h post-transfer

Substrate group	PCR+, substrate/*N* (copy number/µl of sample)	PCR+, container surface/*N* (copy number/µl of sample)	PCR+, flies/*N* (copy number/µl of sample)	VI /IFA+, flies/*N*
Medium only (negative control)	0/2 (ND)	0/2 (ND)	0/10(ND)	0/10
Virus-spiked medium (positive control)	1/2 (2.5 × 10^1^)^a^	2/2 (8.7 × 10^4^)^a^	10/10 (6.3 × 10^5^)^a^	0/10
Virus-spiked milk	2/2 (1.4 × 10^3^)^b^	2/2 (1.1 × 10^5^)^b^	10/10 (5.7 × 10^6^)^b^	1/10

*N*, Total number of samples (wicks, flies or swabs); ND, not detected

^a^SD of virus-spiked medium group substrate, swabs and flies at 24 h post-transfer: 0, 1.8 × 10^4^ and 1.7 × 10^6^, respectively

^b^SD of virus-spiked milk group substrate, swabs and flies at 24 h post-transfer: 1.2 × 10^3^, 7.4 × 10^4^ and 4.3 × 10^6^, respectively

To determine if virus-exposed flies could also transfer virus to the feeding substrate, virus-free substrate solutions were collected from the transfer containers at 4 and 24 h post-exposure. The collected solutions were examined for the presence of viral RNA or infectious virus. After 4 h post-exposure, the virus-free feeding substrate exposed to flies from the virus-positive medium control and the virus-spiked milk groups were all positive for SARS-CoV-2 RNA with low RNA levels of 9.3 × 10^1^ and 1.1 × 10^2^ CN/µl, respectively (Table 2). However, after 24 h post-exposure, only one of two feeding solutions exposed to flies from the virus-positive medium control group was positive for viral RNA with a low RNA level of 2.5 × 10^1^ CN/µL, whereas both samples were positive and the amount of SARS-CoV-2 increased to 1.4 × 10^3^ copies/µL by 24 h post-exposure for the virus-spiked milk substrate (Table 3). Infectious virus was not recovered from any of the substrates incubated with the virus-exposed flies. Together, these results indicate that flies harboring SARS-CoV-2 are capable of transferring virus to virus-free surfaces and feeding substrates; however, the inability to recover infectious virus from these surfaces and substrates suggests that the level of viral contamination is low and below the sensitivity of our virus isolation assay.

## Discussion

*Musca domestica* is the most common fly distributed throughout the world, and due to their gregarious nature and synanthropic predilections, house flies are commonly found associated within or around human habitation [13]. Flies also are indiscriminate feeders that disseminate microbial agents from waste-contaminated breeding habitats to human foods, which in turn can be ingested by unwitting human hosts. SARS-CoV-2 infection causes severe gastrointestinal illness in some infected people, and the virus is shed in their feces [21, 22]. The virus can remain viable in the environment for up to 72 h on smooth surfaces [6, 23] and for days in feces [22] and urine [24].

In order for house flies to mechanically transmit virus, they need to first acquire the virus from a contaminated source, imbibe sufficient amount of virus and harbor the virus in their crop, gut or on their body surfaces; the respective virus must remain stable and viable during this time. In our virus acquisition study, we found that all flies exposed to SARS-CoV-2 in either medium or milk substrates were, after 4 or 24 h exposure times, able to acquire virus. While viral RNA was detected in 100% of flies from the two virus-exposed groups, the flies exposed to virus-spiked milk imbibed more viral RNA than those exposed to the virus-spiked medium control. Interestingly, infectious virus was only recoverable in flies from the virus-spiked milk group (20% of flies after 4 and 24 h exposure times). This difference could be due to the effects of milk on virus stability* versus* culture medium, and is consistent with our results showing increased virus stability in the milk substrate compared to the DMEM growth medium (Additional file 2: Table S1). Similarly, another betacoronavirus, Middle East respiratory syndrome coronavirus, was also found to be stable in animal milk at lower temperatures [25]. The mechanism by which milk enhances the stability of betacoronaviruses is unclear. Interestingly, milk has been shown to keep hepatitis A virus partially stable after heat inactivation [26], suggesting a stabilizing or protective effect of this substrate on viruses, including SARS-CoV-2.

Overall, these studies demonstrated that house flies are indeed capable of acquiring, harboring and transmitting SARS-CoV-2. However, the low level of infectious virus carried by flies limits their capability for SARS-CoV-2 transmission. It should be noted here that only a moderate amount of virus (total of 10^5^ TCID_50_) was used to contaminate 2 ml of feeding substrate. This mimics the real-life situation with up to 7 × 10^8^ RNA copies/swab being shed* via* the upper respiratory tract during the first week of infection [27]. The ability of flies to carry higher amounts of virus will most likely increase with higher virus loads in suitable substrates.

To gain further insights into the potential role of house flies in SARS-CoV-2 transmission, we determined whether flies exposed for 24 h to SARS-CoV-2 could transfer the virus to virus-free environments. Viral RNA was detected on all of the virus-free container internal surfaces and on most of the virus-free substrates exposed to flies that were contaminated either by exposure to virus-spiked medium or virus-spiked milk after short (4 h) or long (24 h) exposure times. Interestingly, flies exposed to the virus-spiked milk substrate transferred more virus to container surfaces and substrates than did flies that were exposed to the virus-spiked medium (Tables 2, 3). This is likely due to a more frequent fly contact with the food-based milk substrate compared to the medium.

Notably, no infectious virus was recovered from any of the samples collected from the surfaces of the transfer containers. This absence of infectious virus could be due to a low amount of virus transferred, the collection method and/or the limit of detection of infectious SARS-CoV-2 virus in the VI assay. Based on the data from the fly acqusition study, the detection of infectious virus is possible when > 10^6^ CN of SARS-CoV-2 RNA/µl are present in the sample (see Tables 1–3). The swab samples collected from transfer containers contained a maximal CN of 10^5^ SARS-CoV-2 RNA/µl, indicating that house flies carry RNA amounts which are on the borderline for containing infectious virus to be detected in our VI assay. A similar observation was made in an another study involving house fly transmission of Newcastle disease virus [16], where house flies harbored an insufficient amount of virus to transmit the pathogen.

Our study has the following limitations. First, we used only one concentration of virus for the contamination of substrates in the studies, whereas in natural settings various concentrations of virus could be accessible by flies, leading to possible dose-dependent effects on pathogen acquisition, persistence and transmission, as shown previously for flies and vector competence for bacteria [28–30]. Secondly, our study did not examine the stability of the virus on the container surface, nor the effect of environmental factors such as humidity and temperature on virus stability. Finally, in our experimental design, we used a lower number of house flies as compared to another study that demonstrated fly transmission of TCV [15]. In the TCV study, it was observed that the infection rate of turkeys increased with higher numbers of virus-exposed flies, indicating that virus transmission by house flies is dependent on the number of contaminated vectors. Future studies involving an* in vivo *vector transmission model with a SARS-CoV-2-susceptible animal species (e.g. mice, hamsters) would be useful to determine the effect of house fly density and duration of exposure on virus transmissibility.

## Conclusions

In the present study, we determined that house flies could readily acquire and harbor SARS-CoV-2 from virus-spiked medium or virus-spiked milk. However, only viral RNA but no infectious virus was recovered from any environmental samples. These data suggest that flies most likely do not play a significant role in transmitting SARS-CoV-2 to humans and susceptible animals. However, the house fly’s ability to harbor SARS-CoV-2 viral RNA for extended periods might offer a potential for its use as a xenosurveillance vector for monitoring SARS-CoV-2 in communities, a technique which more traditionally has been used to survey blood-borne human pathogens by using hematophagous vectors [31, 32].

## Supplementary Information

**Additional file 1: Figure S1.** Indirect Immunofluorescent assay (IFA) for detection of SARS-CoV-2 infected cells.

**Additional file 2: Table S1.** SARS-CoV-2 titers in various test substrates.

**Additional file 3: Table S2.** Acquisition study: Detection of SARS-CoV-2 RNA and infectious virus on inner container surfaces.

## Data Availability

All data generated for supporting the conclusion of this articles included in this article and additional files.

## Abbreviations

CPE: Cytopathic effect
IFA: Immunoflourescent assay
DMEM: Dulbecco’s Modified Eagle’s Medium
TCID_50_: 50% Tissue culture infectious dose
VI: Virus isolation
