# Supplementary material for: Mechanical transmission of SARS-CoV-2 by house flies
Source: Parasit Vectors. 2021 Apr 20;14:214. doi: 10.1186/s13071-021-04703-8 (PMC8056201; doi:10.1186/s13071-021-04703-8)
Supplement: Supplementary file 1 — Additional file 1: Figure S1. Indirect Immunofluorescent assay (IFA) for detection of SARS-CoV-2 infected cells. [file 13071_2021_4703_MOESM1_ESM.pptx]

## Slide 1
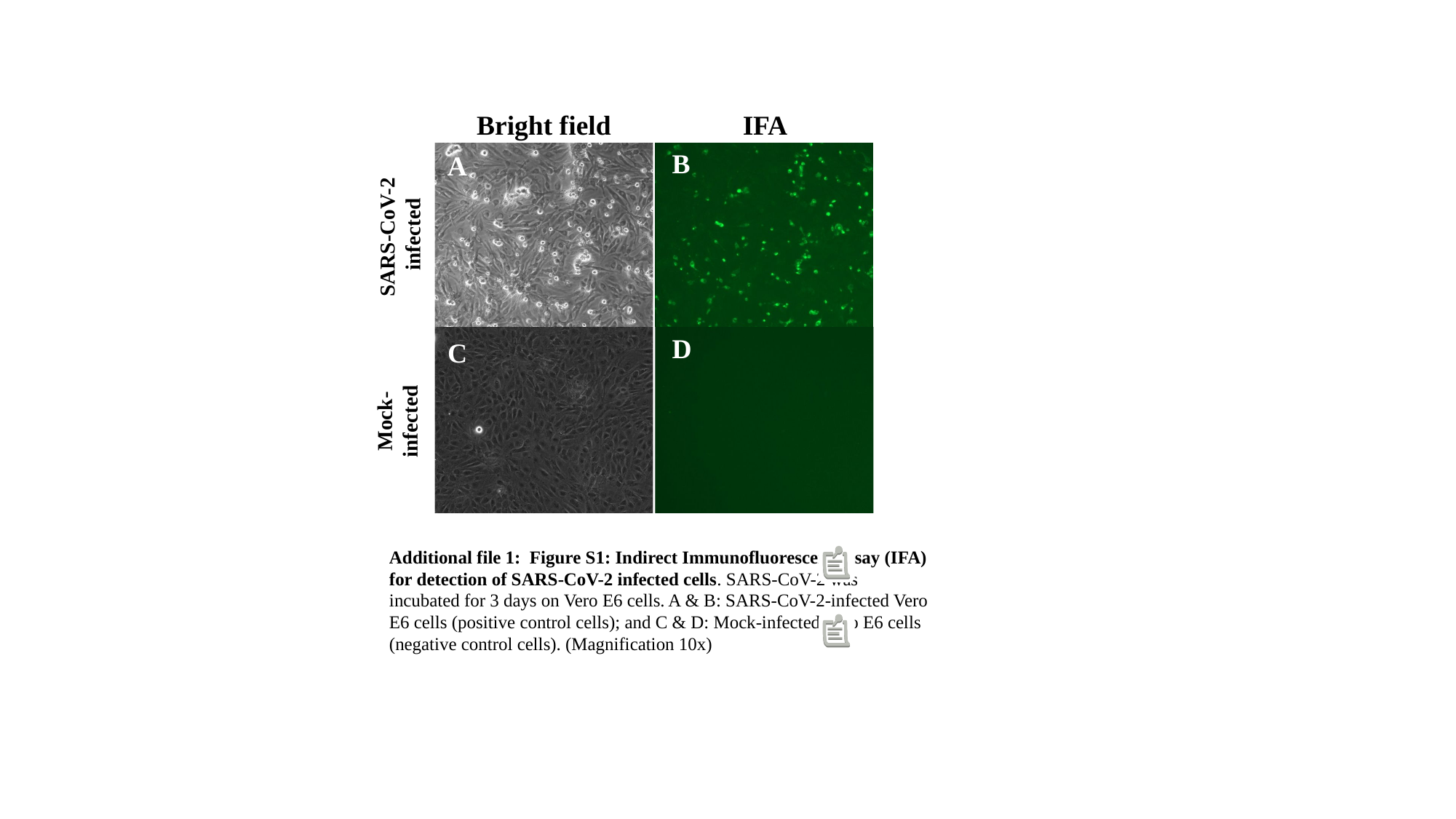

Bright field
IFA
B
A
SARS-CoV-2
infected
D
C
Mock-
infected
C
D
Additional file 1: Figure S1: Indirect Immunofluorescent assay (IFA) for detection of SARS-CoV-2 infected cells. SARS-CoV-2 was incubated for 3 days on Vero E6 cells. A & B: SARS-CoV-2-infected Vero E6 cells (positive control cells); and C & D: Mock-infected Vero E6 cells (negative control cells). (Magnification 10x)
