# Supplementary material for: Mechanical transmission of SARS-CoV-2 by house flies
Source: Parasit Vectors. 2021 Apr 20;14:214. doi: 10.1186/s13071-021-04703-8 (PMC8056201; doi:10.1186/s13071-021-04703-8)
Supplement: Supplementary file 1 [file 13071_2021_4703_MOESM2_ESM.docx]

**Additional file 2: Table S1**. **SARS-CoV-2 titers in various test substrates**.

|  | 0 h | 1 h | 4 h | 24 h |
| --- | --- | --- | --- | --- |
|  | **titer ±SD/ml** | **titer ±SD/ml** | **titer ±SD/ml** | **titer ±SD/ml** |
| Virus-spiked egg yolk solution | 4.4 × 10^3 ^± 1740 | 5.6 × 10^3 ^± 0 | 6.6 × 10^3 ^± 4830 | 4.4 × 10^0 ^± 6 |
| Virus-spiked milk solution | 7.8 × 10^3 ^± 3090 | 4.4 × 10^3 ^± 1739 | 5.6 × 10^3 ^± 0 | 2.5 × 10^3 ^± 969 |
| Virus-spiked sugar solution | 1.0 × 10^4 ^± 10288 | 5.6 × 10^3 ^± 0 | 7.8 × 10^3 ^± 3090 | 1.4 × 10^3 ^± 552 |
| Virus-spiked medium | 1.2 × 10^4 ^± 8549 | 6.2 × 10^3 ^± 1739 | 3.2 × 10^3 ^± 7 | 2.9 × 10^2 ^± 386 |
