# Supplementary material for: Mechanical transmission of SARS-CoV-2 by house flies
Source: Parasit Vectors. 2021 Apr 20;14:214. doi: 10.1186/s13071-021-04703-8 (PMC8056201; doi:10.1186/s13071-021-04703-8)
Supplement: Supplementary file 2 [file 13071_2021_4703_MOESM3_ESM.docx]

**Additional file 3: Table S2.** **Acquisition study: Detection of SARS-CoV-2 RNA and infectious virus on inner container surfaces.**

| **Substrate**      **Group** | **Samples** | **4 h exposure** |  | **24 h exposure** |  |
| --- | --- | --- | --- | --- | --- |
|  |  | **PCR+/N**  **(Copy number** **/µL of sample)** | **VI/IFA+,container surface/N** | **PCR+/N** **(Copy number** **/µL of sample)** | **VI/IFA+, container surface/N** |
| **Medium only** **(negtive control)** | container swabs | 0/2 (ND) | 0/2 | 0/2 (ND) | 0/2 |
| **Virus-spiked medium** **(positive control)** | container swabs | 2/2 (1.8 × 10^6^)^a^ | 1/2 | 2/2 (9.7 × 10^3^)^a^ | 0/2 |
| **Virus-spiked milk** | container swabs | 2/2 (1.1 × 10^5^)^b^ | 0/2 | 2/2 (2.6 × 10^5^)^b^ | 0/2 |

N= total number of swabs; ND= not detected

^a^ Standard deviation (SD) of virus-spiked medium group swabs at 4 h and 24 h exposure: 2.4 × 10^6 ^, and 1.1 × 10^4 ^, respectively.

^b^ SD of virus-spiked milk group swabs at 4 h and 24 h exposure: 8.5 × 10^4 ^, and 2.5 × 10^5 ^, respectively.
